# Decoding the Role of Glycans in Malaria

**DOI:** 10.3389/fmicb.2017.01071

**Published:** 2017-06-09

**Authors:** Pollyanna S. Gomes, Daniel F. Feijó, Alexandre Morrot, Celio G. Freire-de-Lima

**Affiliations:** ^1^Departamento de Imunologia, Instituto de Microbiologia, Universidade Federal do Rio de JaneiroRio de Janeiro, Brazil; ^2^Instituto Gonçalo Moniz, Fundação Oswaldo Cruz (Fiocruz)Salvador, Brazil; ^3^Instituto Oswaldo CruzFiocruz, Rio de Janeiro, Brazil; ^4^Instituto de Biofísica Carlos Chagas Filho, Universidade Federal do Rio de JaneiroRio de Janeiro, Brazil

**Keywords:** malaria, glycobiology, carbohydrate-targets, *Plasmodium falciparum*

## Abstract

Complications arising from malaria are a concern for public health authorities worldwide, since the annual caseload in humans usually exceeds millions. Of more than 160 species of *Plasmodium*, only 4 infect humans, with the most severe cases ascribed to *Plasmodium falciparum* and the most prevalent to *Plasmodium vivax*. Over the past 70 years, since World War II, when the first antimalarial drugs were widely used, many efforts have been made to combat this disease, including vectorial control, new drug discoveries and genetic and molecular approaches. Molecular approaches, such as glycobiology, may lead to new therapeutic targets (both in the host and the parasites), since all interactions are mediated by carbohydrates or glycan moieties decorating both cellular surfaces from parasite and host cells. In this review, we address the carbohydrate-mediated glycobiology that directly affects *Plasmodium* survival or host resistance.

## Introduction

Malaria is caused by a protozoan of the *Plasmodium* genus, which belongs to the *Apicomplexans* phylum, an obligatory parasite (Kishore et al., 2013). Only in the last two decades has there been a significant reduction in malaria caseloads, and, according to the latest World Health Organization (WHO) records, cases have dropped significantly, i.e., from 262 million in 2000 to 214 million in 2015 (WHO, 2015). It is still a serious condition, however, noteworthy when compared to other pathologies of equal importance worldwide, due to its high prevalence (Binns and Low, 2015). Efforts toward the elimination of malaria involve the extermination of its vectors, and parasites and universal access to prevention (diagnosis and earlier treatment); this includes the discovery of new drugs, to deal with the high number of drug-resistant strains (Tanner and Hommel, 2010; Feng et al., 2016).

The first malaria drug treatments began with the use of quinine as the active ingredient (Parola and Miller, 2002). During World War II, a quinine-derivative, chloroquine, was widely used and treated as a “top secret” (Loff and Cordner, 1999; Skvara, 2004). Chloroquine was associated with many benefits (low cost, efficacy, and safety) (Kofoed et al., 2003; Savarino et al., 2006). Quinine-based drugs were replaced by Artemisinin derivatives and other drugs; and the use of Artemisinin-based Combined Therapies (ACT) is now recommended (Visser et al., 2014; Watsierah and Ouma, 2014; Pousibet-Puerto et al., 2016) to eliminate the blood phases, since in the exoerythrocytic phase (asymptomatic phase), there are no obvious symptoms for early treatment (Imrie et al., 2007).

Most of these drugs are still in use today in different doses, depending on the infective species and host background (Achan et al., 2011). Resistance is usually accompanied by a range of genetic diversity, and a high level of polymorphism, crucial to spreading these infective parasites but also after the widespread use of drugs, the first resistance-cases have appeared, and it seems that drugs have an “expiration date” this has also been observed in different malaria-infected patients in different regions, such as Thailand and Papua New Guinea (Cui et al., 2003; Brito and Ferreira, 2011). For example, *Plasmodium falciparum* shows high antigenic variation, with more than 60 coding variations of the *P. falciparum* erythrocyte membrane protein 1 (*Pf* EMP-1), directly related to the virulence and lethality of the infection of this species (Arnot and Jensen, 2011). On the other hand, may present variations in the merozoite surface protein MSP-3α is a multi-gene family important in *P. falciparum* and *Plasmodium vivax*, acting as “decoys” for antigenic diversity during RBCs invasion (Rice et al., 2014). CSP genes or circumsporozoite protein (CSP) in sporozoite surface, thrombospondin-related anonymous protein/sporozoite surface protein 2 (TRAP) or else in *P. vivax* apical membrane antigen 1 (AMA1) in ectodomain and C-terminal region of MSP-1 as a immunodominant antigen that was studied with recombinant protein (MSP119) as a novel potential vaccine (Rocha et al., 2017) and liver stage antigen (LSA1) also studied in malaria vaccine approaches (Pichyangkul et al., 2008). Thus, these and many more key proteins at each *Plasmodium* stage open up the “branches” for studies of this type of interactions, as seen in glycobiology.

## Carbohydrates in malaria: approach for potential drug target discovery

Glycosaminoglycans (GAGs) are abundant in both host and parasites; they are composed of basic units of carbohydrates that rearrange themselves in various ways, changing function and location (Griffin and Hsieh-Wilson, 2013). Glycobiological approaches investigate the influence of these carbohydrates on host-parasite binding interactions, such as glycolytic enzymes that are adequate in predicting a good understanding of parasite metabolism and glycosylation of malaria proteins. The first evidence about sugars mediatING the parasite-red blood cell invasion was cited by Miller et al. (1977). Experiments determinated that O-linked oligosaccharides, such as NeuNAc and GalNac, were found in high concentration (20 mM) and inhibited the parasite intracellular invasion in RBCs (Pasvol, 1984). Other sugars such as Gal (β1-3) GalNAc disaccharide associated with glycophorin was more inhibitory in the same context (Hermentin et al., 1984).

Thus, these tools give support to studies currently in development in this regard. In addition, some pathogen-associated molecular patterns (PAMPs) consist primarily of carbohydrates structures, although these are not yet well known or understood in malaria parasites (Hoving et al., 2014). However, recently, the most discussed PAMPs are GPI anchores, haemozoin, and immunostimulatory nucleic acid motifs (Gazzinelli et al., 2014).

Other crucial receptor crucial, that requires specific receptor-ligand interactions to RBC invasion and cytoadherence in malaria, is Duffy-binding-like domains (DBLs). In *P. vivax* and *P. knowlesi*, parasites invade RBC exclusively through the DARC receptors (Duffy antigen receptor for chemokines). However, in *P. knowlesi* DBL domain (Pkalpa-DBL) to due a immune pressure they seems development a evasion strategy to run away, mapping to opposite surface of the DBL.

Spitzmuller and Mestres (2013) addressed the design of a generation of new antimalarials drugs. A major challenge is to identify *P. falciparum* proteins, among million possible combinations that can be targeted at the same time by the just one drug. In their studies, they analyzed databases and to identify drugs with multi protein targets, because the drugs until now supported specific protein targets, which in a few time allows the parasite to mutate only at this target reaching. Unlikely, Artemisinin which is regarded as a multi-target drug, maintaining as a new generation drug and which is advocated throughout malaria treatment (Spitzmuller and Mestres, 2013).

Still regarding innate immunity, there are two major families of pattern recognition receptors (PPR) predominantly expressed by cells of innate immune system are TLRs and C-type lectin receptors (CLRs). CLRs are important for the immune response against parasites, and in some studies example has been observed that, for example, CLRs are related with cerebral malaria in mice infected by *Plasmodium berghei*, and CARD9 is upregulated, but CARD9^−/−^ mice were not protected from infection, suggesting that the CARD9 receptor influences infectivity by the plasmodium but in its absence (as demonstrated in knockout animals) does not prevent the disease from occurring (McGuinness et al., 2003). Another study conducted with *P. chabaudi* demonstrated that the mannose receptor C type 2 (MRC2) increased with parasitemia, but toll-like receptors and sialoadhesin decreased in contrast to other MRCs (1 and 2), and that decreased with parasitemia in *P. yoelii*, suggesting the importance of lectin-receptors in the development of mounting of the immune response (Rosanas-Urgell et al., 2012).

Studies regarding parasite sugar supply demand has increased in the last decades, given that *Plasmodium* parasites require a high sugar demand to replicate. These parasites also show the ability to manipulate vector behavior to ensure survival, including increased sugar seeking, although it is unclear how this manipulation affects vector-plant interactions and sugar uptake (Nyasembe et al., 2014). Parasite manipulation in search of sugar supplies has been described as established at the moment of vector infection. *Plasmodium* present in the bloodstream require glucose, which crosses the plasma membranes and enters the parasite cytosol (Coppi et al., 2005; von Itzstein et al., 2008; Bertolino and Bowen, 2015; Swearingen et al., 2016). The parasites are able expose the RBC hexose transporter to facilitate sugar nucleotide uptake, allowing *Plasmodium* to biosynthesize certain glycans for maintenance (Cova et al., 2015). After the release of the sequence in the PlasmoDB, database facilitated the search for tools in interventions in this receptor for therapeutic purposes. The hexoses receptor (PfHT) has been widely studied, because its decrease implies in the lower supply of glucose to parasites and causing the plasmodium elimination (Bahl et al., 2003).

The surface of infected red blood cells (RBCs) are rich in glycophorins and *Plasmodium* possesses some proteins like erythrocytes binding-like (EBL) and reticulocyte binding-like (Rh) protein families that recognize them, playing a critical role in attachment in invasion (Davidson and Gowda, 2001; Salinas et al., 2014), Studies demonstrated that glycophorins as play crucial role in *Plasmodium* invasion, in absence of glycophorins A relatively resistant to the invasion in red blood cells (Pasvol, 1984). Other portion of glycophorin A has sialic acid residues, which is known as EBA-175 (175 kDa), and it mediates binding of *P. falciparum* to RBCs. A part of this, EBA-175 is highly conserved and rich in cysteine, is referred to as F2 (PfF2) and it has receptor binding sites that have been studied as a possible recombinant protein in malaria vaccines trials (Pattnaik et al., 2007).

In Tham et al. (2015) the cytoplasmatic tails of these proteins were phosphorylated *in vitro* and blocked RBC invasion, evidencing the importance of these proteins for invasion (Tham et al., 2015).

In addition to studies on the interactions between the parasite and carbohydrates in RBCs, it has been demonstrated that blood type (ABO, Lewis, Duffy, and others surface antigens) influences erythrocyte parasitism, with certain types more susceptible to *Plasmodium* infection (Cooling, 2015). Studies indicate that individuals of blood group A are highly susceptible to *P. falciparum* induced-malaria, while blood group O has been shown to be protective against complicated cases (Fischer and Boone, 1998; Lell et al., 1999). The CSP and TRAP domains on the sporozoite that mediate the adhesive contact with the sulphated glycoconjugates on the surface of hepatocytes allow plasmodium invasion to the bloodstream. Thus, these proteins are extremely important for the parasite, since it is from the entry in the hepatocytes that the cycle begins. Taking this into account, these same proteins have been studied extensively, including in the manufacturing of anti-malarial vaccines, such as RTS,S (Coppi et al., 2005; Swearingen et al., 2016). Despite having obtained good results in treating mice with anti-CSP, it has been verified that, in the absence of this protein, the cycle of hepatocyte invasion continues normally, since after invasion CSPs are less expressed, while other proteins become highly expressed (Bertolino and Bowen, 2015).

As the *Plasmodium* parasite uses sugar-requirements to ensure survival, approaches with drug-targeting carbohydrates have increasingly been proposed as possible treatments. Regarding *in vitro* studies, Plaimas et al. (2013) investigated a database of genetic information from *Plasmodium* to try to decipher which points of the proteins expressed could be future of therapeutic targets. 22 potential targets, refined the search by removing false positives, leaving only 5 targets, among them, glutamyl-tRNA (gln) aminotransferase and with a known inhibitor of this transferase 6-diazo-5-oxonorleucine (Don). The tests were carried out and the growth of the parasite decreased both *in vitro* and *in vivo* in Swiss mice, despite the side effects related to the dosage (Plaimas et al., 2013). Similar results have recently been observed with DON in experimental cerebral malaria mice models, although this compound has shown inhibitory effects by blocking CD8+ T-cell effector function, which is the highest cause of mice death (Gordon et al., 2015). On the other hand, other studies indicate that mice mortality was attenuated due to GPI anchors, not T cells, since several literature reports indicate the importance of glycophosphatidylinositol (GPI) anchors for the success of *Plasmodium* infection (Naik et al., 2000). CSP have also been associated to GPI, which have a canonical domain in the COOH portion, although this has not yet been demonstrated (Coppi et al., 2005).

Of significance, people living in endemic areas are more resistant to malaria, due to the production of antibodies against GPI anchors (Vijaykumar et al., 2001).

In addition to inhibitors that hinder this type of parasite-host interaction, mice immunized with the glycan moiety of GPIs were able to produce anti-GPI antibodies to prevent progression to cerebral malaria (Schofield et al., 2002). It has been reported that a microbial polyssacharide, Gellan Gum (GG), containing a sugar moiety produced by the *Sphingomonas* (*Pseudomonas*) *elodea* bacterium, strongly inhibited parasite invasion; this also inhibits growth (strains 3D7 and Dd2), demonstrating that “natural” sugars can also contain *Plasmodium* effects (Recuenco et al., 2014). In human malaria, successive pregnancies contribute to resistance against *Plasmodium*, which mediates binding to chondroitin sulfate A (CSA) in the placenta through the VAR2CSA protein (Salanti et al., 2004; Gamain et al., 2005); this leads to accumulation of *Plasmodium* parasites in the placenta, resulting in severe clinical consequences for both mother and child (Resende et al., 2008), such that the investigation of this interaction is a viable target for vaccines (Clausen et al., 2012; Fried and Duffy, 2017). Through all this information about the various receivers composed by sugar and the interactions required these reinforce the importance of deciphering the nature of glycan functions in malaria in order to improve approaches for predicting drug-target interactions for this complex.

## Author contributions

PSG, DFF, AM, and CGF-de-L wrote the paper. All authors read and approved the final version of the manuscript.

### Conflict of interest statement

The authors declare that the research was conducted in the absence of any commercial or financial relationships that could be construed as a potential conflict of interest.
